# “The real ethernet”: The transnational history of global Wi-Fi connectivity

**DOI:** 10.1177/14614448221103533

**Published:** 2022-06-23

**Authors:** Maria Rikitianskaia

**Affiliations:** London School of Economics and Political Science, UK; Regent’s University London, UK

**Keywords:** 802.11, standardization, telecommunications, transnational history, Wi-Fi networks, wireless

## Abstract

Wi-Fi is an integral and invaluable part of our media practices. Wireless networks are blended into our media environment and, in terms of infrastructural importance, have become comparable with electricity or water. This article offers a new transnational perspective on the underexplored history of IEEE 802.11 standards by focusing on the tensions between the United States and Europe in terms of development trajectories of wireless technology. The goal is to analyze the standardization of wireless networking through a transnational lens and to contribute to enhanced understanding of the global proliferation of Wi-Fi technology. Four particular aspects of the transnational development of Wi-Fi technology are discussed: the rivalry between US and European standards, the constitutive choice to focus on data transmission, radio spectrum availability, and the peculiarities of network authentication.

Wi-Fi is an integral and invaluable part of our media practices. Wireless networks are blended into our media environment and, in terms of infrastructural importance, have become comparable with electricity or water. Today, nearly every home in the developed world has its own Wi-Fi network. In 2018, there were 169 million public Wi-Fi hotspots worldwide. By 2023, 628 million public Wi-Fi hotspots are predicted to be available (Cisco, 2020: 13). New residential and office buildings are being constructed with built-in broadband Internet outlets in storage cupboards for installation of a Wi-Fi router. Telecommunication operators offer Wi-Fi extenders to cover every corner of a house with a Wi-Fi network. The recent circumstances of the COVID-19 pandemic and resulting lockdowns have only magnified the crucial importance of Wi-Fi in our everyday lives. It revealed expectations to have a wireless connection at home that is comparable with that at the office, the school, and the university in terms of speed, reliability, and convenience.

Despite the phenomenal proliferation of Wi-Fi networks, very few scholarly works have scrutinized the historical formation of Wi-Fi standards and their worldwide spread (Lemstra et al., 2011; Thomas et al., 2021). Wi-Fi has become such an integral part of contemporary daily routines that it goes unnoticed in most theoretical reflections on transforming mediascape. As Adrian Mackenzie (2010) put it, Wi-Fi networks have been characterized by “insignificance and blandness” (p. 3). Thus, we know relatively little about how Wi-Fi started to occupy such an essential part of our media practices.

This article offers a new transnational perspective on the underexplored history of Wi-Fi standards. The goal is to analyze the standardization of wireless networking through a transnational lens and to contribute to enhanced understanding of the global proliferation of Wi-Fi technology. The article focuses on the tensions between the United States and Europe in terms of development trajectories of wireless technology. The research questions addressed in this work are as follows: What was the wireless market like at the time Wi-Fi development began in the 1990s? What constitutive choices were made by engineers in the process of Wi-Fi standardization? What specific imaginaries of wireless connectivity were considered during the formation of Wi-Fi, and how did they affect the process of Wi-Fi development? Following a social constructionist approach to the history of technology, this article discusses the rivalry between different European and US wireless local area network (LAN) standards, which held contrasting visions about the future of wireless.

Based on original archival research into primary sources, this article reveals that the global success of Wi-Fi was the result of meticulous efforts to construct a standard that would withstand a competition with the related European standards by strengthening the data transmission capabilities instead of the usual focus on voice communication.

## Standardization of wireless as a social construction

The Wi-Fi standard is the leading technology among a myriad of wireless communicative formats. Nevertheless, few technologies, except for running water and electricity, are as integrated as Wi-Fi with contemporary expectations of normally functioning infrastructure and day-to-day experience.

What kind of technology is Wi-Fi? Unlike a smartphone, a dishwasher, or a combustion engine, it is difficult to point to something and call it “Wi-Fi.” We might have routers and wires in our homes, but in institutions and public places, these instruments are largely hidden from view. Strictly speaking, Wi-Fi is a standard for packet radio networks, which were commonplace long before standardization under the name “Wi-Fi.” The idea behind a packet radio network is simple: to send packets of data via radio waves, thus combining existing radio broadcasting techniques with Internet-based networking. The name “Wi-Fi” refers to a very particular kind—or family—of packet radio network: IEEE 802.11 standards (802.11a, 802.11b, 802.11n, among others). The standards were developed in the 1990s in the United States; the name IEEE 802.11 stands for the 802.11 working group at the Institute of Electrical and Electronics Engineers (IEEE). This working group explicitly focused on Wi-Fi belongs to a larger collection of working groups at the IEEE dedicated to working on Internet connectivity standards.

This article focuses on the social construction of the Wi-Fi technology standard. According to the social construction of technology (SCOT) approach, it is important to deconstruct the interpretative flexibility, describe the technological artifact’s social construction, and explain its construction process in terms of relevant social groups (Bijker, 2009). In the 1990s, Wi-Fi was precisely at a stage of interpretative flexibility, which means, in the words of Pinch and Bijker (1984), that “different social groups have radically different interpretations of one technological artefact” (p. 423). Using the terms of Jonathan Sterne (2003), Wi-Fi could also be characterized by its plasticity or the malleability of wireless and the malleability of computing and connecting to the Internet in those years. Thus, following the SCOT approach, this article focuses on the debates, negotiations, and conflicts through which the Wi-Fi standard was co-constructed, growing out from the interpretative flexibility into the point of stabilization (Pinch and Bijker, 1984). Through understanding of those debates, this article also reveals the sociotechnological visions on wireless future (that drove those discussions and debates) that can also be considered as wireless imaginaries (Jasanoff and Kim, 2009).

The scholarship has mostly studied the present of Wi-Fi rather than its past. Wi-Fi networks have been researched in their role of building community networks and “bottom-up infrastructures” (Crabu and Magaudda, 2017; Forlano, 2009; Meinrath, 2005; Shah and Sandvig, 2008); they have been studied through their public dimension (Hampton and Gupta, 2008; Hampton et al., 2010; Powell, 2008; Tapia and Ortiz, 2010) and particular attention has been paid to their license-exempt position in the radio spectrum (Werbach, 2003).

The historical approach to wireless networks so far has been limited to very few scholarly works. The most comprehensive historical account of the formation of Wi-Fi standards is the research of historians of technology Wolter Lemstra and John Groenewegen with contributions by Vic Hayes, himself the chairman of the 802.11 working group. This research was primarily based on interviews with and the recollections of the engineers (2011). The marketing side of Wi-Fi proliferation has also been studied by Mackenzie (2006). The social and cultural consequences of Wi-Fi integration in our everyday practices have been overviewed in the recent book by Thomas et al. (2021). Some historical overviews of the creation of Wi-Fi standards can also be found scattered in various works on the history of networks, standardization processes, and Internet history (e.g. Abbate, 1999; Balbi and Magaudda, 2018; Greenstein, 2012; Mackenzie, 2010; Milgrom et al., 2011; Rikitianskaia and Balbi, 2020; Russell, 2014). Thanks to these accounts, there is some fragmented understanding of the history of the Wi-Fi networks through its peculiar entanglement with the history of computing, the Internet, networks, and digital media.

The experiments with wireless networks for computers and digital devices date back to the 1970s, when the Hawaiian islands were connected without cables to ALOHAnet, the pioneering computer networking system (Lemstra et al., 2011). ALOHAnet inspired other, sporadic, fragmented but mostly successful projects of connecting computers with packet radio networks. They became even more frequent from 1985, when the US Federal Communications Commission (FCC) released the industrial, scientific, and medical (ISM) radio bands for unlicensed use. Although these bands initially were intended for purposes other than telecommunications, the following decade witnessed introduction of several wireless networks that used these bands, such as Token-Ring and WaveLAN.

As computers became increasingly portable and interconnected, the standard of wireless networking was necessary to develop the wireless industry further on a global scale. Standardizing the protocols is essential for developing the communication industry because it helps different equipment suppliers produce devices capable of interoperability (DeNardis, 2011). Other standard protocols designed in the 1970s and 1980s, such as those for addressing and routing data, clock synchronization, and local area networking Ethernet, laid the foundation for the Internet up to this day. Thus, in 1990, the IEEE formed the 802.11 Working Group for Wireless LANs to standardize wireless networks, and in 1997, the committee issued the original IEEE 802.11 standard.

The literature on standardization demonstrates that the process of creating a standard is profoundly political and can be very controversial (Bowker and Star, 1999; DeNardis, 2009, 2011; Yates and Murphy, 2019). Standards set the rules for others to follow (Busch, 2011). The development of standards is a representative example of the social construction of a technological system, which, in the words of Thomas Hughes (1989), is “both socially constructed and society shaping” (p. 51). A standard is based on existing knowledge and experience in engineering but formulates certain specifications for future equipment and defines the use of the technology. As Andrew Russell (2014) noted, “standards committees do not make technology, rather they make agreements about technology” (p. 19). The standardization process affects the vision of the technology and favors specific approaches and techniques over others. “Standardization had obvious benefits,” as Janet Abbate (1999) put it, “but the choice of any particular protocol as an international standard would also create winners and losers among the creators and users of network technology” (p. 148).

The case of Wi-Fi standardization indeed created some winners and losers in the wireless industry. Before the 802.11 standards, early experiments in wireless LANs could not overcome the challenge of bringing costs down. As Greenstein (2012) put it, they “hit this wall like a textbook cliché throughout the 1990s” (p. 19). The 802.11 standards boosted the manufacturing of a standardized product at a relatively low cost. The “winners” were the companies NCR and Lucent, both de facto representing AT&T.

The proliferation of Wi-Fi did not happen gradually but rather was a result of targeted attempts of the wireless industry, represented by numerous computing actors. It required years of normalization before Wi-Fi would become an integral part of our media use (Morley, 2019). In 1999, Apple Computer Company introduced a consumer 802.11b device, the Airport, which allowed the sharing of Internet access and files between multiple computers at a rate up to 11 Mbit/s. The same year, the IEEE 802.11b specification pioneers formed a Wireless Ethernet Compatibility Alliance (WECA) (later renamed “Wi-Fi Alliance”) to facilitate the global spread of Wi-Fi standards (Mackenzie, 2006). The Alliance comprised more than a dozen technological companies, such as Apple, Samsung, LF, Motorola, and Microsoft. As an intelligent marketing move, the name Wi-Fi was coined to resemble the acronym Hi-Fi (high fidelity), a generic term used to indicate high-quality audio technologies. In 2003, the official magazine of the International Telecommunication Union (ITU) spotlighted the rapid success of Wi-Fi with an article titled, “Wi-Fi Takes the (Communication) Sector by Storm” (*ITU News*, 2003: 29).

Thus, histories of computing, Internet, and standardization provide a good basis for understanding and researching the history of Wi-Fi. Wi-Fi networks are at the center of what Fortunati (2017) calls the “historical relationship between the fixed-line Internet and the mobile phone” (p. 184). Along with mobile networks and other signals, Wi-Fi networks have become a key media-technological element of “wirelessness” (Mackenzie, 2010) and of significantly enhanced digital connectivity (Camponovo et al., 2014). Even geolocation is now effected not only by a satellite-based radio navigation system, the Global Positioning System (GPS), but also by cell phone and Wi-Fi triangulation (Ceruzzi, 2018).

Wi-Fi thus constitutes an important part of our media-technological environment and intertwines with other media and communication networks that evolved through similar processes of standardization and commercialization. The historical importance of the subject, however, warrants much more than these fragmented and scattered appearances.

This article seeks to build upon this historical research of the Wi-Fi standards by offering new insight into this history through a transnational perspective. Drawing on science and technology studies, the article mainly focuses on the constitutive choices made in the formation of the standard over the years, while it was still in a stage of interpretative flexibility. Furthermore, following Internet scholars (Bory, 2020; Brügger et al., 2017; Schafer, 2015), this article specifically helps to expand our understanding of Internet histories in its complexity of rival projects, intertwined paths, and various networking projects within diverse political, economic, social, and cultural contexts.

## Sources

This research project addresses the transnational exchange of knowledge, people, goods, and information that influenced the standardization of wireless networks, following media and communication scholars who researched global and entangled media histories (e.g. Christensen, 2013; Iriye and Saunier, 2009; Van der Vleuten and Feys, 2016). This helps to uncover how standard of wireless networks was formulated specifically for global endeavors.

The aim of this historical analysis is to identify the key socio-technical decisions that underlie the 802.11 standard, as well as the network of actors and organization that involved in those regulatory agreements. The research mostly focuses on the key challenges that Wi-Fi standardization faced, as they repeatedly appeared on agendas of numerous meetings and discussions.

The analysis is based on a large and heterogeneous corpus of national and transnational primary sources, of the period from 1985 to 2003. The historical sources for this research consist of three groups of documents. The first set of sources consists of documents of the IEEE 802.11 working group’s primary documents, such as minutes, agendas, reports, and other documents exchanged among its members. The analysis of these sources enabled us to identify the key issues regarding wireless networks and the context of the discussions. The second set of sources consists of the documents from the international standardization body International Telecommunication Union (ITU) (including the minutes of the international telecommunication conferences and respective conventions, as well as 433 monthly issues of the ITU Journal), and the news and press releases of the European standardization body, the European Telecommunications Standard Institute (ETSI). This set of sources helped analyze the global agenda of wireless networking standardization and to grasp how the IEEE 802.11 standard was regarded from the European perspective. Finally, the research considered reports and press releases overviewing the progress of the wireless standards, published by the relevant telecommunication companies such as, Cisco Systems, Apple Inc., British Telecom, and others.

## Findings

The findings bring us to two significant conclusions that can contribute to media and communication studies as well as science and technology studies.

First, the article presents two contrasting imaginaries of the wireless future and discusses how they impacted the creation of Wi-Fi standards. These imaginaries dominated the US and European trajectories of wireless development and were characterized by the opposition of data transmission versus voice communication, respectively.

Second, the article reveals that the history of Wi-Fi was intertwined with other related media. Rather than being acknowledged exclusively as a part of computing history, Wi-Fi should be seen as part of telephonic history and radio studies. Wi-Fi is not only a critical infrastructure for our contemporary media practices but also an inter-technological and inter-media artifact with historical implications.

The following paragraphs present the historical evidence for these two main findings and discuss in detail four particular aspects in the transnational development of Wi-Fi technology: rivalry between US and European standards, the constitutive choice to focus on data transmission, radio spectrum availability, and the peculiarities of network authentication.

### The battle of wireless standards

From the beginning of the formation of the IEEE 802.11 working group, the engineers agreed to consider the European market for any potential wireless standard. Rick Albrow representing the privately held UK company Symbionics, referred to European initiatives in the following way: “It may be wise to watch what they do” (IEEE P802.11, 1990b: 3).

At that time, at least seven different international standards bodies were developing various wireless standards.^1^ Governments worldwide encouraged different experiments on the “transfer of LAN data over an air interface” (Black, 1991: 4). The most peculiar project was the Digital European Cordless Telephone (DECT), which had been in development by the European Telecommunications Standard Institute (ETSI) from the late 1980s. In the early 1990s, the project changed its name to Digital European Cordless Telecommunications (DECT), which indicated a corresponding change of perspective: from specifically telephonic to telecommunicative, including wireless LANs. Originally, DECT was scheduled to be issued by the end of 1991, and thus it had to be out in the market even sooner than IEEE 802.11.

Another competitor on the European scene was the Global System Mobile (GSM) also by ETSI, which provided a wide area of coverage as a cellular network. The GSM was first introduced in Finland in 1991 for full-duplex voice telephone, later included data communications, and over time evolved into 3G and 4G (Kammerer, 2010). DECT had more focused coverage (no more than a single building in some cases); however, it offered a higher transmission quality. GSM, on the contrary, had extensive coverage but a lower quality of transmission. Retrospectively, we know that GSM achieved success as a cellular phone network, while DECT has been used for cordless phones. Nevertheless, it is essential to acknowledge that both were initially seen as rivals to Wi-Fi and were considered possible wireless standards.

The rivalry between the wireless standards established a competitive arena for the development of the 802.11 standard, which aligns very well with other accounts of Internet networking history in the 1990s. Andrew Russell noted that in the 1990s, the Internet witnessed scaling and commercialization problems (Russell, 2014). The IEEE 802.11 standard was created in the context of scaling and commercialization too—as a commercially viable product to be marketed, promoted, and sold. Not all members of the working group agreed with this approach. As James (Jim) Neeley, then Vice Chairman of IEEE P802.11, representing IBM LAN Systems Design, said, “We are developing a standard. Not deciding to manufacture to a specific market.” He illustrated how the standard was supposed to have universal applications by comparing it with a Swiss army knife: “It is a very general-purpose tool, you will be surprised at the usage that tool is put to” (IEEE P802.11, 1991a: 18).

Despite this criticism, the discussions on defining the Wi-Fi standard in the 1990s were still led by marketing goals and competition with other standards. The decisions made in this regard did not pursue technical needs but also took into account the downsides and advantages of competitive wireless standards. A similar battle over interconnectivity was happening in the arena of European research networks in the early 1990s. Valerie Schafer (2015) called it a “battle of the protocols” (p. 221), meaning that despite the shared need for interconnection, it was problematic for European countries to link their existing national networks, such as JANET in Britain, DFN in the Federal Republic of Germany, and SURFNET in the Netherlands. Similarly, the rivalry with other technical bodies became an important factor for the social construction of the IEEE 802.11 standard. One could even call it the battle of wireless standards.

The members of the working groups were debating the pros and cons of the competing technology standards, seeking to cover all the possible loopholes with their product. Simon Black (working for the UK company Symbionics, but also “representing DECT” as working group meeting minutes indicate) noted on the progress of DECT formulation:

DECT is a written standard with some features that we may not want to reinvent. It may be a competitor. DECT has done competent marketing. DECT is not infallible; it is important that this group understand and not make the same mistakes. (IEEE P802.11, 1991a: 22)

Curiously, despite being part of an American organization, the IEEE 802.11 working group was composed mainly of European technical professionals, with the IEEE 802.11 chairman Vic Hayes, representing NCR Systems Engineering, being from the Netherlands. Thus, the working group was in an advantageous position: working in Europe and having social networks there, it could design a standard addressed to both the United States and Europe. Even the application for radio bands allocation indicated that Europe was ahead of the United States in creating wireless standards. Thus, competition with Europe was a defining factor for the development of the wireless standard (IEEE P802.11, 1992a: 10).

Apart from Europe, the working group IEEE 802.11 was also concerned with “harmonization” with Canada, Australia, and Japan. Some of the IEEE 802.11 meetings took place in Canada and engineers considered different experiments with wireless conducted in the country. In Australia, they foresaw no problems with the introduction of a new wireless standard in the spread spectrum band, which seemed “very liberal” (see more on spread spectrum in “Radio spectrum availability” section) (IEEE P802.11, 1991b: 6). The working group also decided to follow Japan closely in its wireless development (IEEE P802.11, 1992a: 10). Even though no report on Japanese technologies was available for some time, it was known that Japan planned to introduce a “connectionless service” at 2.4 GHz (IEEE P802.11, 1992c: 3). Developments were occurring in the area of wireless LAN for Personal Computers, designed by Fujitsu, which was similar to GSM modems (IEEE P802.11, 1991b: 7). Japan was crucial in this process because the Japanese digital mobile telephone system included sharing specifications with a US trade delegation (Wilkus, 1991: 5).

### Constitutive choice to focus on data transmission

To compete in the global market, the IEEE 802.11 standard had to offer certain distinctive features that would help it stand out from other wireless network standards. The most important and distinctive feature for IEEE 802.11 became its exclusive focus on data transmission rather than voice communication. Retrospectively, this focus on data does not seem all that surprising, but it was something new and radical for the time. Long-lasting debates and negotiations shaped this decision. These discussions were an example of what Balbi and Fickers called “techno-diplomacy,” which is characterized “by strategic actions, tactical manoeuvres among all actors involved and, generally, require a high degree of both technical knowledge and diplomatic skills by the negotiating parties” (Balbi and Fickers, 2020: 1–2).

Telecommunication carriers initially saw data communication as an extension of telephony and did not expect computer-to-computer interaction over public data networks. In general, mobile telephony was central for national telecommunication market strategies in the 1990s (Abbate, 1999; Bory, 2020; Kammerer, 2010). So vividly present in Internet history, this focus on voice transmission also impacted wireless networking history. Long before the creation of the Wi-Fi standard, experiments with wireless LANs had already been embroiled in controversy concerning data and voice transmission. When Robert Kahn and his colleagues in 1975 experimented with a pioneering wireless system, PRNET, in the San Francisco Bay area, it was entirely designed for voice transmission. Even packet switching was considered beneficial only to the extent that it made voice transmission more efficient and less vulnerable to eavesdropping (Kahn et al., 1978). There was no need to connect computers at that time. As Kahn (1990) later recalled,

In 1973, mainframe computers were multi-million dollar machines that required air-conditioned computer centres. You weren’t going to connect them to a mobile, portable packet radio unit and carry it around. (p. 25)

Only in the 1990s did Kahn’s experiment become a significant point of reference for the engineers who were developing wireless LANs for data transmission (IEEE 802.11, 1997: 3).^2^

Even in the early 1990s, the FCC was still not considering data transmission as a significant technological advancement. Instead, the FCC was more concerned with developing “new personal communication services (PCSs)” focused on voice communication, more specifically “advanced cordless telephone and portable radio systems for personal use” (FCC, 1990: 1). These services were supposed to “free individuals from the constraints of the wireline public switched telephone network and enable them to communicate when they are away from their home or office telephone” (FCC, 1990: 1). In other words, the idea was to develop a mobile phone network. It was noted that this interest was global, and in particular, the United Kingdom was “especially active in the area of PCSs,” as it had allocated a spectrum for an advanced digital cordless telephone technology, referred to as CT-2 (FCC, 1990: 2).

A remarkable idea of developing networks in public spaces was also suggested. A proposal was made to establish a so-called “telepoint service.” In the United Kingdom, four providers were licensed to set up base stations in public places, such as airports, shopping centers, and restaurants. Within the base station range, a subscriber to the service could call by using their personal CT-2 handsets (FCC, 1990: 3). This idea to organize small networks within specific public spaces predates the use of Wi-Fi in public areas, even though it focused on voice transmission over data transmission. The first descriptions of the use of IEEE 802.11 inherited that vision. As early as 1990, the use of Wi-Fi was specified “in buildings such as offices, financial institutions, shops, malls, small and large industry, hospitals, outdoor areas such as parking lots, campuses, building complexes and outdoor plants and storages” (IEEE 802.11, 1990a: 1). The only difference was that 802.11 offered data transmission, instead of just voice communication.

In shaping the 802.11 standard, the working group members slowly abandoned the original focus on voice transmission suggested by the FCC. The market for the wireless standard dictated a different imaginary for the wireless future. The IEEE 802.11 standard targeted another customer, different from the user with a personal handset.

The first customers for the Wi-Fi standard came from the retail industry: cash machines. Cash machines, unlike mainframes, had to be more portable in order to respond to the needs of merchandising. Department stores were constantly rearranging their collections, thereby also moving their transaction terminals. Rewiring them was massively inconvenient, and therefore connection via radio waves was essential. It seems only logical that the most important actor in the development of Wi-Fi was the National Cash Register (NCR) Corporation, known for manufacturing and selling the first mechanical cash register in the late 19th century. No voice communication was needed—the cash registers did not speak to each other.

The computer industry had also been involved in the development of Wi-Fi—more specifically, Apple Computer Company presented comments on the design of wireless at the first meetings of IEEE 802.11 and emphasized the importance of data transmission (IEEE 802.11, 1990b). Moreover, Apple promoted data transmission to the FCC Commission. In 1992, for instance, on the en banc hearing of the FCC, the chairman spoke about cable TV, cellular, and telephone as crucial developments for the “future competitiveness of the nation,” and Apple added the case for data to the list (IEEE P802.11, 1992b: 4). It is no wonder, then, that the Wi-Fi standard, once issued, was very rapidly introduced into Apple products. Marina Mazzucato (2013) ingeniously demonstrated that Apple profited many times from state-funded technological innovations, later using them in their products. The Wi-Fi case is very similar: Apple influenced the creation of the wireless standard on the regulatory level for its benefit by insisting on data communications. This helped Apple to employ techniques used in the IEEE 802.11 standard, such as the previously secret spread spectrum technique, therefore again following the same pattern: profiting from the results of public-sector research rather than investing in private-sector investigation.

Thus, the focus of data transmission became a distinct characteristic of the IEEE 802.11 compared with European technologies, such as DECT or GSM. In Europe, data transmission was important as well, but secondary to voice communication. As Simon Black summarized at the IEEE 802.11 group meeting, “DECT is a voice-oriented standard that is working hard to incorporate data, as apposed [*sic*] to 802.11 which seems to be a data-oriented standard that is working hard to incorporate voice” (IEEE P802.11, 1991a: 15). Europe was seen as a possible customer for the Wi-Fi spread, as, in Europe, “the potential market demand for cordless LAN products remained largely untapped, primarily due to a lack of spectrum and standardization” (Black, 1991: 4).

Furthermore, the 802.11 working group had a critical advantage. Their liaison with the IEEE 802, which had previously developed the Ethernet standard dominating the market of wired networks, had an impact on the design. The engineers could consider designing wireless LANs as complementary to the wired networks, consequently ensuring their smooth integration. The Wi-Fi standard has even been called “the real ‘ethernet,’” thus alluding to the wordplay of “ether” as in radio ether (IEEE P802.11, 1990a: 6).

Slowly but steadily, voice transmission slowly fell out of view of the 802.11 working group. As Bruce Tuch from NCR Systems Engineering noted, “Voice is nice—we should have the hooks for voice, but it should not have priority, and we will drop it if it is too costly” (IEEE P802.11, 1991a: 11). The 802.11 working group directed all efforts toward data transmission, and in the long run, those efforts were rewarded.

### Radio spectrum availability

Today, contemporary communications rely on the entanglement of invisible radio waves coming through a “mediatized air” (Rikitianskaia, Balbi, Lobinger, 2018), in the form of mobile phone coverage, Bluetooth technologies, and Wi-Fi networks. All these radio-based technologies depend on the radio bands allocated to them by the governmental and inter-governmental organizations responsible for radio spectrum management. Thus, the Wi-Fi standardization directly depended on the geopolitics of the radio spectrum. To secure the global potential for Wi-Fi growth, IEEE 802.11 had to consider radio spectrum management in different countries and regions.

The main regulatory body for the radio spectrum on the international level is the ITU. However, its global approach does not cover all radio spectrum management and national bodies must still regulate most parts of communication services. In particular, France and Italy were seen as problematic countries for allocating frequencies for Wi-Fi. The IEEE Project 802.11 could have been a failure merely due to the unavailability of the spectrum: “there is no guarantee that the resulting spectrum requirements will be accommodated throughout Europe” (Black, 1991: 4). Accordingly, the IEEE 802.11 had to find an appropriate way to make a Wi-Fi standard viable and functional on a global market.

The European standards had undisputable advantage regarding the radio spectrum, as most of European nations pursued the vision of a shared radio spectrum, meaning a united space for radio and mobile communications (IEEE P802.11, 1990b: 3). With the creation of the Memorandum of Understanding (MoU), European nations agreed to set aside specific bands for DECT, CT-2, and GSM that targeted cordless telephone communication from slightly different angles. The approach of shared communications also aligned well with the idea of open networks in the European space (Henrich-Franke, 2020). To produce a competitive standard, it was thus reasonable for the IEEE to consider the same bands as in Europe (IEEE P802.11, 1992a: 3). However, some of the European requirements concerning the spectrum were not advantageous for wireless data transmission. A discussion arose in 1992 regarding these bands, Nathan Silberman from California Microwave Inc. argued that more bandwidth would be required for data transmission (IEEE P802.11, 1992a: 8–9). In other words, what worked for cordless telephones did not work for Wi-Fi.

Thus, the complex radio spectrum management in a variety of countries led to the development of IEEE 802.11 standard in an area of spectrum that required little regulations and modifications. The working group aimed at finding appropriate radio bands that would be available in most of the regions, would not be overcrowded with other devices, would be already used for communication purposes, and, moreover, will be wide enough for the data communication. The ideal solution was a 2.4 GHz band. It was available in the United States because it was opened as an ISM band. It was available in Europe for low-power communication devices, but the direct contenders of Wi-Fi were using other bands: DECT standard was using the 1.88–1.9 GHz, and European Conference of Postal and Telecommunications Administrations (CEPT) decided to favor 5.2 GHz for wireless communication network (IEEE P802.11, 1992b: 4). Furthermore, the band was used for wireless communications in some other countries, such as aforementioned Japan. Developing data communication on this band was thus a convenient decision.

Furthermore, the group chose to use the previously secret military technology of the spread spectrum. It allowed for the use of different frequencies, in contrast to the more traditional uses of the radio spectrum. Traditional wireless media, such as radio and television broadcasting, demanded the allocation of a particular part of the radio spectrum for their use only. On the contrary, spread spectrum technology made it possible to not occupy the radio waves completely. One way of implementing this technology is called frequency-hopping spread spectrum. The idea is to transmit information with a constant change of frequencies. Both the transmitter and receiver know the algorithm for switching frequencies and can thus catch the entire message. Ease of use is coupled with delays in the transmission of information every time there is a jump. Another method, also frequently used in Wi-Fi, is the direct sequence spread spectrum. Its implementation is much more complicated: The technique multiplies the data by a pseudorandom spreading sequence at a much higher bit rate than the original data rate. The message then resembles broadband white noise, which is typically detected and eliminated by conventional radio devices and does not cause interference. Conversely, conventional radio devices do not interfere with the broadband signal because they operate on a narrow frequency. Therefore, this military technology, designed to conceal communication and reduce interference, was ideal for creating a cable-free environment.

Overall, these circumstances called for the creation of a wireless standard that would be easy to introduce under any radio spectrum policy. Therefore, decisions were made to ensure that the easy commercialization of the wireless standard culminated in a technology that was essentially license-exempt. Wi-Fi continues to expand today in the “gray area” of international regulations, causing a kind of “radio revolution” (Werbach, 2003). The consequent low cost and easy set up to deploy, maintain, and scale Wi-Fi networks for ordinary users helped to connect many digital devices at home, such as PCs, tablets, smartphones, TV sets, printers, cameras, baby monitors, and others. It is not surprising that the Wi-Fi hotspots experienced a surge in numbers simultaneously with the spread of 3G networks and they offered similar mobile connectivity at a lower cost (Lemstra and Hayes, 2009). Unlike the cell phone systems monopolized by the telecommunication providers, the Wi-Fi connectivity required no licenses, permission, and fees and had a liberating character.

### Open authentication

In recent decades, Wi-Fi networks have been massively criticized for their vulnerability. Due to the competition that the standard has had to face on the market, specifications were established for open networks from the very beginning.

The competitors, such as the DECT standard, emphasized the security of the network over its accessibility. One of the features of the DECT standard was “restricted access to the network by authentication and security of data during transmission by encryption” (Black, 1991: 4). Security was considered an important feature that made DECT “a serious contender in the European cordless data market” (Black, 1991: 5). Of the potential cordless LAN users, 70% cited the security of radio links as a crucial factor that could affect the uptake of cordless LAN technology. Thus, the authentication procedures of DECT were designed to “prevent unauthorized access to the network and encryption of transmitted data to guard against eavesdropping” (Black, 1991: 5). Consequently, DECT became a standard that allowed point-to-point telephone communication, which was incorporated into cordless telephones. Therefore, the protection of DECT communication was high.

The Wi-Fi networks, on the contrary, were not that strictly closed. The 1995 draft of the Wireless Access Method and Physical Layer Specifications discusses wireless networks in relation to the wired LANs (Bagby, 1995): “The media impacts the design” (p. 2). This ironically sounds nearly synonymous to McLuhan’s (1994) famous slogan: “Media is the message.” Wireless is more dynamic than comparable wired LAN connections: It does not have observable boundaries and it is unprotected from outside signals. The area’s concept is problematic by itself because for wireless, “well-defined coverage areas simply do not exist” (p. 7).

Substantial attention was paid to the issue of the security of the network. The section “Security Services” in the initial draft did not just undergo significant changes but was entirely rewritten after a meeting in May 1995. The IEEE 802.11 defined two authentication schemes: shared key and open system. The shared key mechanism refers to the use of a password while connecting to a Wi-Fi network, one which is distributed independently from the wireless connection (e.g. written on the back of the router). The open system, as the name suggests, is a system without any password. Despite having created this open mechanism, the document of specifications underscored several times that it was not recommended. It said, “If desired, an 802.11 network can be run without authentication. 802.11 cautions against this as it may violate implicit assumptions made by higher network layers” (Bagby, 1995: 17).

Along with the authentication mechanism, the standard also included a privacy service created to prevent eavesdropping. This service was responsible for encrypting messages using the WEP algorithm. However, as noted, this algorithm was not designed “for ultimate security” but rather to be “at least as secure as a wire” (Bagby, 1995: 19). Once again, the vision of Wi-Fi was dominated by the idea of interconnections to wired networks. Interestingly, the working group “specifically recommended against running an 802.11 with privacy but without authentication”; however, it still introduced this option. The draft specified that “While this combination is possible, it leaves the system open to significant security threats” (Bagby, 1995: 43).

The main reason for leaving the open system authentication standard was interoperability and the urge to facilitate connectivity. This openness went hand in hand with the rhetoric of openness that Andrew Russell (2014) described as “ideological commitment to entrepreneurship, technological innovation, and participatory democracy” (p. 1). This rhetoric was very prominent in creating community Wi-Fi networks and other Wi-Fi-related initiatives in the amateur radio community in the 2000s (Dunbar-Hester, 2009; Hampton and Gupta, 2008; Middleton and Crow, 2008). However, open authentication necessarily entailed easy access to the network at the cost of security. The standard has also allowed expansion of the supported authentication schemes in the future, which indeed was the case in the 2000s.

Within years of the vast proliferation of open Wi-Fi networks, the ephemeral risk to security became a palpable problem. Both public and private open networks provided access to everyone, including unauthorized users. Thus, the illegal use of the Internet had to be restricted. The only way of doing so was to track and “log” individual users’ Internet connections, thereby tracing them afterward to limit their liability. The subsequent years of Wi-Fi development witnessed several modifications to the standard and transformations of the wireless practices that valued protected connection over an easy access. The consequence of this delayed implementation of additional security measures is conceptualized as “a chilling Internet use,” in Robin Mansell’s words (Mansell, 2012: 128). Over the years, this security problem in public Wi-Fi networks was addressed by the broad introduction of so-called “captive portals,” which provide web-based authentication, typically via user registration by email or phone number. Often, captive portals are used for marketing and commercial communication purposes. The spread of this method, which is not a part of the 802.11 standard but is instead an accessory to network installation, is indicative of a problem in maintaining Wi-Fi networks. These different lifetimes of public Wi-Fi networks, that is, first without and later with captive portals, could be seen as different “maintenance regimes” (Russell and Vinsel, 2018).

## Conclusion

The proliferation of wireless in everyday life routines was foreseen as early as the beginning of the 1990s. In 1991, Mil Ovan from Motorola Inc. predicted,

Over the next 20 years, society will witness a significant “wireless evolution” in both personal and professional communications, and change the way we conduct our lives at home, on the road, and at work. (p. 2)

This prophecy did not just foresee the global wireless “evolution” but also attributed it to the development and proliferation of the IEEE 802.11 standard. The 802.11 standard was explicitly designed to dominate the wireless market and beat other standards globally. The members of the 802.11 working group aimed to create seamless wireless connections via radio waves, ironically calling it “the real ‘ethernet’” (IEEE P802.11, 1990a: 6).

This article presents findings from the historical analysis of the creation of the Wi-Fi standard. In the 1990s, the uses and roles of Wi-Fi were as of yet undecided. Essentially, Wi-Fi was at a stage of interpretative flexibility in that it was amenable to a variety of views. Not only was the wireless standard undecided, but parallel trajectories were not yet developed. There were different jurisdictions, various concerns about the technology, and numerous wireless industry actors, each of whom had different ideas about the future of wireless.

This article discussed how certain decisions made with regard to 802.11 specifications were based on the global competitiveness of the technology. More precisely, the article outlined how the standard design was oriented toward creating a viable and strong alternative to European wireless standards. The 802.11 standard was focused on data transmission rather than voice transmission, thereby permitting higher-speed connections comparable with those of wired LANs. However, the problem with entering the global market was the availability of the radio spectrum, especially considering that European countries had signed an MoU to share frequencies for their wireless standards. Thus, when targeting the global market with radio-based technology, the 802.11 working group had to consider the availability of the radio spectrum worldwide. The spread of spectrum technology and the exploitation of the ISM bands helped to solve this issue. Furthermore, to differ from European standards, the 802.11 standard had to offer open user authentication at the expense of security. This pursuit stood in stark contrast to that of DECT, which emphasized the safety of the network over its accessibility. This ultimately led to significant problems with Wi-Fi security and the consequent creation of additional safety measures in the 2000s—it also slowed the proliferation of Wi-Fi worldwide.

These findings lead us to two significant conclusions that, hopefully, make an essential contribution to the history of computing, Internet, media and communication studies, and science and technology studies.

First, this article demonstrated two drastically different narratives with respect to the development of wireless standards in Europe and the United States. Inherent to these narratives are two opposing and competing visions of the future of wireless. European telecommunication organizations targeted mobile and voice communication and were ahead of their US colleagues for some time. Due to certain advantages in the geopolitical situation in Europe, such as the agreement to create a unified space for mobile communication, the United States had few opportunities to compete in the wireless market unless it could find some alternative route—and it did. Wireless networking for computers and personal devices was, indeed, that alternative vision of the future of wireless, one which was not initially planned by US authorities but instead emerged from computer industry players familiar with the European backstage. Although it might seem surprising in retrospect, data transmission was not initially envisioned, only becoming a priority to permit the 802.11 standard to compete on the wireless scene. The focus on data transmission became a constitutive choice in developing the Wi-Fi standard, one that led to its consequent success. The two different narratives of European and American telecommunications to offer voice and data transmission, respectively, can be scrutinized to deepen our understanding of the history of the computer industry and mobile communications.

Second, the development of the Wi-Fi standard highlights the central theme in the history of technologies and telecommunications: their relationship with the radio and the telephone. The history of Wi-Fi is also deeply intertwined with radio spectrum management and other radio-based technologies, thereby making it a part, broadly speaking, of radio studies (Rikitianskaia and Balbi, 2020). Like many technologies that were inspired or driven by innovations in telephone research (see, for example, Sterne, 2012), Wi-Fi emerged from the notion of using telephones in public spaces and both simultaneously and oppositely approaching telephones as a medium of voice. Along with many other media technologies that have been inspired or driven by telephone research innovations (see, for example, Sterne, 2003, 2012, on sound reproduction), Wi-Fi is part and parcel of “the telephonic history of technology” (Balbi and Berth, 2019). The Wi-Fi networks have evolved as a contender for the existing business of telecommunication providers, with a focus on telephone systems.

Moreover, this article drew our attention to the evolution of the uses of Wi-Fi networks. Initially, Wi-Fi networks were developed to connect cash machines. This fact enriches our understanding of the wireless networking development and draws attention to the under-researched link between money and media (Swartz, 2020). Then, the Wi-Fi networks quickly attracted the interest of the computer industry. They were thereafter used to connect computers and laptops to the Internet and, later, to smartphones. In this evolution, we can see the prominence of the telephone in the history of wireless connectivity. Originally developed to differ from phone networks, Wi-Fi was, eventually, ultimately put into the service of mobile phones, as phones today are a primary locus of data. Thus, these findings on the origins of Wi-Fi help to bridge Internet history, the history of mobile communication, radio studies, and other fields of media and communication research.

Today, we are witnessing new transnational tensions concerning the further technological development of Wi-Fi technology. As in 1985, when the FCC released the ISM bands, in April 2020 the FCC released a 6 GHz band for unlicensed use, lending to Wi-Fi its first major boost in decades. This band provides a fourfold increase in the radio spectrum to Wi-Fi routers and wireless devices, giving them more bandwidth and lowering interference. New devices incorporating this spectrum range are branded under the name “Wi-Fi 6E.” Considering the geopolitics around 5G mobile phone networks, this innovative version of Wi-Fi is strategically important for the United States to maintain a dominant position in the wireless industry. As this article showed, transnational controversies have surrounded wireless advancements from the very beginnings of Wi-Fi and will continue to accompany the technology as connected devices increase in number and wireless coverage continues to expand.

## Notes

1. Moreover, some of the standards could be developed in one body but then moved to another—for instance, Global System Mobile (GSM) was established by the European Conference of Postal and Telecommunications Administrations (CEPT) but later moved to ETSI.

2. Kahn’s experiment with packet radio networks still played an essential role in media history at large, as it allowed Kahn to acknowledge the importance of interconnecting heterogeneous networks, which later compelled him to develop the Internet program.
